# APOBEC3G-Depleted Resting CD4^+^ T Cells Remain Refractory to HIV1 Infection

**DOI:** 10.1371/journal.pone.0006571

**Published:** 2009-08-10

**Authors:** Francesca R. Santoni de Sio, Didier Trono

**Affiliations:** Global Health Institute, School of Life Sciences, “Frontiers in Genetics” National Center of Competence in Research, Ecole Polytechnique Fédérale de Lausanne (EPFL), Lausanne, Switzerland; Institut Pasteur, France

## Abstract

**Background:**

CD4+ T lymphocytes are the primary targets of HIV1 but cannot be infected when fully quiescent, due to a post-entry block preventing the completion of reverse transcription. Chiu et al. recently proposed that this restriction reflects the action of APOBEC3G (A3G). They further suggested that T cell activation abrogates the A3G-mediated block by directing this protein to a high molecular mass complex.

**Methodology/Principal Findings:**

In the present work, we sought to explore further this model. However, we found that effective suppression of A3G by combined RNA interference and expression of HIV1 Vif does not relieve the restrictive phenotype of post-activation resting T cells. We also failed to find a correlation between HIV resistance and the presence of A3G in a low molecular complex in primary T cells.

**Conclusions/Significance:**

We conclude that A3G is unlikely to play a role in the HIV restrictive phenotype of quiescent T lymphocytes.

## Introduction

CD4+ T lymphocyte cells are the primary *in vivo* targets of HIV1. However, early studies made it clear that, both *in vivo* and *in vitro*, these cells are refractory to HIV1 infection when in a quiescent state. Indeed, after viral entry into resting T cells, reverse transcription is initiated but fails to reach completion [1]. CD4+ T lymphocytes isolated from peripheral blood (PB) become permissive to HIV1 replication once activated through the T cell receptor or stimulated *in vitro* with cytokines such as interleukin-2 (IL-2), IL-7 and IL-15. Accordingly, exposure to cytokines secreted *in vivo* in the microenvironment of lymphoid tissues might explain why resident naïve CD4+ T cells are permissive to the virus [1]–[5].

The differential expression of cellular factors in resting and activated T cells has been proposed as an explanation for these observations (reviewed in [6], [7]), but conclusive evidence for their role has been lacking. Recently, apolipoprotein B mRNA editing enzyme catalytic polypeptide 3G (APOBEC3G – A3G) has been put forth as the post-entry restriction factor responsible for HIV1 restriction in resting CD4+ T cells [8]. A3G was initially discovered as potent anti-HIV1 factor countered by the HIV *vif* gene product [9]. Several studies demonstrated that this and selected other cytidine deaminases, if expressed in *vif*-defective (ΔVif) HIV1 producer cells, are packaged into outgoing retroviral particles and lethally edit nascent viral reverse transcripts once the virus enters target cells [10]–[12]. In recent work, Chiu et al. reported that A3G, in resting CD4+ cells, is found in an enzymatically active form within a low-molecular-mass (LMM) complex, and based on RNAi-mediated depletion experiments claimed that it was as such responsible for the HIV1 restriction observed in these targets [8]. In contrast, in activated CD4+ cells, A3G was noted to be present in high-molecular-mass (HMM) ribonucleoprotein complexes as a catalytically inactive protein, possibly explaining why it did not have such an effect. It was further suggested that, in quiescent T cells, A3G could restrict both wild-type and ΔVif HIV, since it was acting before integration and viral protein production, and since only insignificant amounts of Vif are present in incoming virions [8].

Stimulated by this interesting claim, we decided to explore the mechanisms of A3G-mediated post-entry HIV1 restriction in resting CD4+ T cells. However, we report here that we found no evidence of an antiviral effect of the cytidine deaminase in such a setting.

## Results

**Figure 1:**
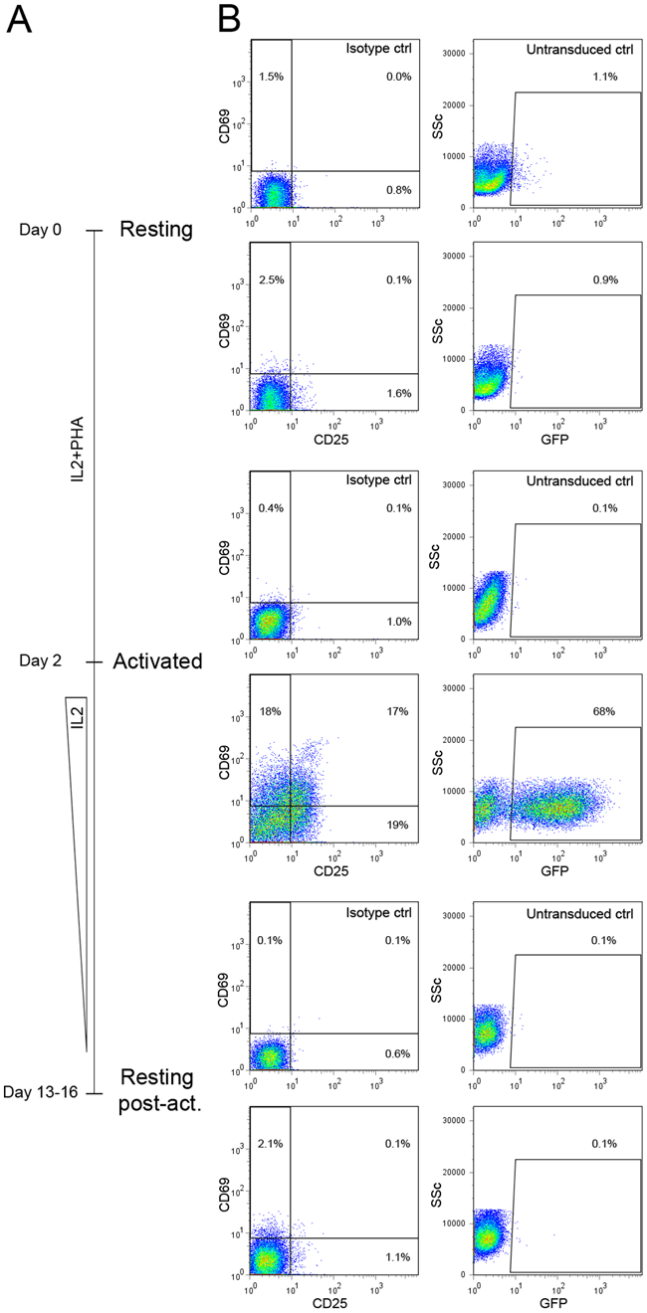
Experimental approach. A, CD4+ cells were purified from human peripheral blood (resting), exposed to high-dose IL2 and PHA for two days (activated) and then maintained for 11–14 days in low-dose IL2 (resting post-act.). B, the three cell populations were analyzed for expression of activation markers expression (CD25, CD69) (left) and GFP production 3 days after infection with a GFP-containing HIV1 derivative.

In their work leading to the proposal that A3G is responsible for restricting HIV in quiescent CD4+ cells, Chiu et al. down-regulated the protein by siRNA tranfection [8]. In order to facilitate subsequent mechanistic studies, we decided to use an alternative model, in which activated CD4+ T lymphocytes were transduced with lentiviral vectors carrying effector sequences, and analyzed for HIV1 permissiveness once back to a resting and restrictive state. For this, CD4+ T cells were purified from human PB (resting cells), stimulated by IL2 and phytohemagglutinin (PHA) (activated cells) and led to rest again by decreasing IL-2 concentration in the culture medium (resting post-activation cells) (Figure 1a). To validate our model, we first compared the level of activation and susceptibility to infection of the three populations, which were analyzed by flow cytometry for the presence of activation markers and for GFP expression following exposure to a GFP-expressing HIV reporter virus. As expected, activated T cells expressed high levels of the activation markers CD25 and CD69, and were susceptible to HIV infection (68% of GFP+ cells). In contrast, resting post-activation cells expressed negligible levels of activation markers and were fully restrictive to HIV infection (0.1% of GFP+ cells), as were un-activated resting cells (1% GFP+ cells) (Figure 1b).

**Figure 2:**
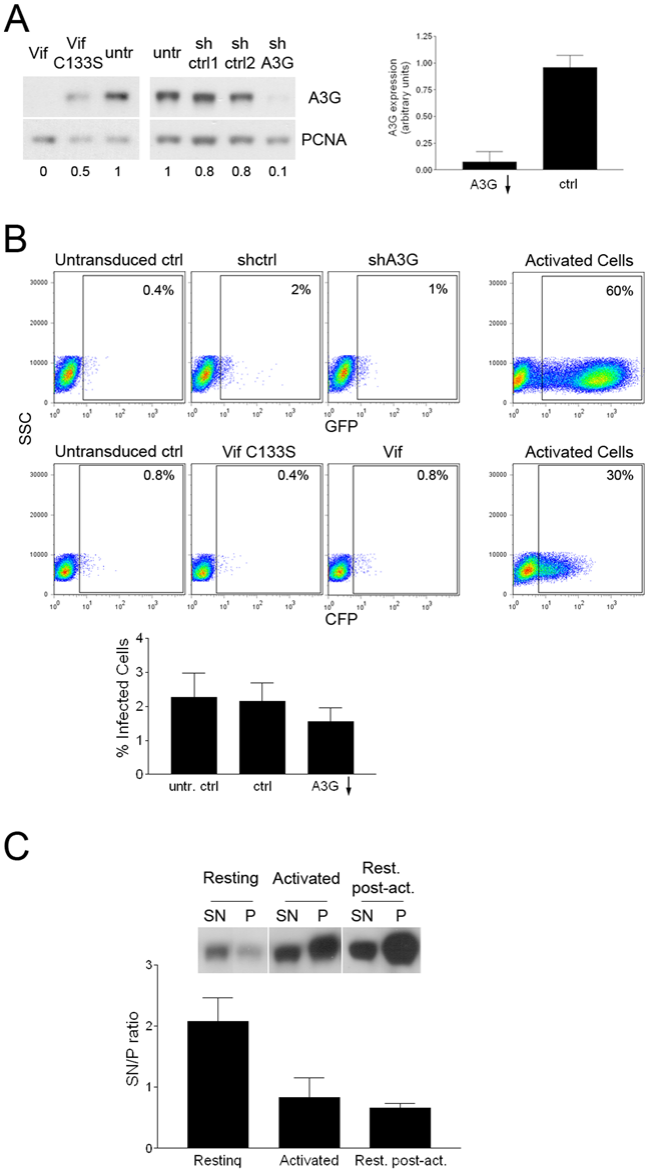
A3G knock-down does not relieve HIV restriction. Activated CD4+ were transduced with HIV vectors expressing Vif, Vif C133S, shRNA against A3G (shA3G) or mismatched shRNA (sh ctrl), or left untransduced (untr), and let to rest. A. Resting post-activation cells were analyzed by Western blot for A3G expression. Left panel, two representative experiments out of four performed are shown. Right panel, average and SD of A3G expression level normalized to PCNA expression level in the Vif or shRNA to A3G transduced cells (KD) and control cells (ctrl) are shown (n = 4). B. Resting post-activation cells expressing shA3G or Vif (A3G KD), shCtrl or Vif C133S (ctrl), or not previously transduced (untr. ctrl) and activated cells were infected with GFP- or CFP-expressing HIV reporter virus and analyzed by flow cytometry. Upper and middle panels, two representative experiments out of four performed are shown. Lower panel, average and SD of the percentage of infected cells in all the experiments are shown (n = 4). C. Resting, activated and resting post-activation (Rest. post-act.) cells were subjected to ultracentifugation and supernatant (SN) and pellet (P) from each population were analyzed by Western blot with A3G specific antibody. Upper panel, one representative experiment out of three performed is shown. Lower panel, average and SD of the ratio between expression level of A3G in the supernatant and the pellet (SN/P ratio) are shown (n = 3).

In order to knock down A3G expression, we used a combined protein- and RNA-based approach. We took advantage of the HIV-1 Vif protein, which counters A3G by triggering its proteasomal degradation [13], [14]. We transduced activated T cells with lentiviral vectors expressing wild type Vif or, as a control, the C133S Vif mutant, which still interacts with A3G but fails to induce its degradation [15]. We verified the functionality of wild-type Vif by demonstrating that it could counter the effect of A3G on ΔVif HIV-1 in activated T cells, in contrast to the C133S Vif mutant (data not shown). Also, we transduced activated T cells with lentiviral vectors expressing shRNA directed to A3G (shA3G) or, as a control, a mismatched shRNA. We then tested A3G levels of expression in the resting post-activation cells. Western blot analysis with A3G specific antibody showed a reduction of the A3G protein level of 90% on average when cells were transduced with Vif- or shA3G-expressing vectors (Figure 2a).

Then, we evaluated the effect of A3G knock down on the susceptibility to HIV infection of resting post-activation T cells. We performed single-round infection experiments with VSV-G-pseudotyped HIV reporter virus expressing GFP or CFP. T cells expressing Vif or shA3G, or Vif C133S or the control shRNA, were exposed to HIV reporter virus and analyzed 3 days later for fluorescent protein expression. Despite the efficient down-regulation of A3G, resting post-activation T cells were still as restrictive to HIV infection (1.6% GFP+ cells on average, 2 experiments with shA3G- and 2 with Vif-expressing cells) as untransduced or control-transduced cells (2.3% and 2.1% GFP+ cells, respectively; average of 4 experiments) (Figure 2b). This was not due to a lack of infectivity of the reporter virus, because when used at the same multiplicity of infection (MOI) in activated T cells it efficiently induced stable GFP expression (Figure 2b). Based on these data, we conclude that A3G does not mediate HIV restriction in resting T cells.

Finally, to evaluate whether A3G was present in a low molecular mass complex in resting post-activation CD4+ T cells, as previously observed in resting CD4+ T cells [8], we performed high speed centrifugation. This assay allows separating cytosolic unbound A3G in the supernatant (SN) fraction form the higher molecular forms of A3G in the pellet (P) fraction [16]. Resting, activated and resting post-activation T cells were subjected to high speed ultracentrifugation, and SN and P fractions from each sample were subjected to Western blot analysis with an A3G-specific antibody. As previously shown [16], resting cells showed a 2–3 fold higher SN/P ratio when compared to activated cells, confirming that A3G in resting T cells is prevalently in a low molecular mass form that shifts to a high molecular mass complex when cells are activated. In contrast, the SN/P ratio in resting post-activation T cells was comparable to the SN/P ratio in activated cells, indicating that in these cells A3G is mostly present in a high molecular mass complex (Figure 2c). Thus, there is no correlation between the presence of A3G in a LMM complex and a post-entry restriction of HIV1 infection.

## Discussion

The resistance of resting T cells to HIV1 infection has been the focus of numerous studies. While most have concurred to demonstrate that this phenotype represents a post-entry bock to reverse transcription, the mechanism at play and its cellular mediators have remained elusive [1], [2], [17], [18]. Recently, Chiu et al. proposed that A3G was responsible for the observed restriction [8]. However, this claim was challenged by Kamata *et al.* [19], who did not observe any relief of HIV blockade following transfection of A3G-specific siRNAs in quiescent CD4+ T cells, that is, following the very same protocol as Chiu et al. Here, we studied the question using a different experimental setting, albeit one in which HIV1 restriction in quiescent T cells is fully revealed. In order to down-regulate A3G, we transduced activated CD4+ T cells with shRNA/Vif-expressing vectors and analyzed their susceptibility to HIV1 infection once they had returned to a quiescent state. In spite of achieving equal if not higher degrees of A3G down-regulation compared to Chiu et al., we found no relief of HIV1 restriction in resting cells. We can only speculate on the bases of the discrepancy between the two sets of results. However, it is worth noting that transfection can be toxic in primary T cells, leading to cell death, which in turn can induce activation in the surviving population. Although Chiu and colleagues did not find any increase in activation markers in transfected cells, it cannot be excluded that their manipulation triggered the cells to exit from the G_0_ to the G_1_ phase of the cell cycle. This progression, not monitored in their study, has been shown to suffice for T cells to become permissive to HIV infection [20]. It could thus have accounted for their finding of a higher HIV susceptibility of siRNA-transfected T cells, although this effect would have had to be specific to the A3G and not control siRNAs.

We cannot formally exclude that the restrictive phenotype observed in resting post-activation cells has some distinctive features compared with its counterpart in un-stimulated cells, even tough the two settings are phenotypically similar. Still, our data together with results recently published by Kamata *et al.* [19] indicate that HIV restriction in resting T cells is not mediated by A3G. Furthermore, our demonstration that A3G associates with low and high molecular mass complexes, respectively, in unstimulated and resting post-activation CD4+ cells, without differential impact on HIV1 permissiveness, invalidates the proposed model of an inhibition specifically mediated by LMM-associated A3G [8]. The mechanisms of HIV restriction in quiescent T cells as well as the potentially distinct functions of the two forms of A3G thus remain to be fully elucidated.

## Methods

### Cells

Human peripheral blood was obtained after written informed consent by healthy donors from the Centre de Transfusion, Centre Hospitalier Universitaire Vaudois, Lausanne, Switzerland. Work with human samples was approved by written consent by the Ethical Committee of the Centre Hospitalier Universitaire Vaudois, Lausanne, Switzerland, and conducted according to the principles expressed in the Declaration of Helsinki.

Mononuclear cells were isolated from human peripheral blood by Ficoll-Paque™ Plus (Amersham Biosciences) density centrifugation and subjected to negative magnetic selection with CD4^+^ T Cell Isolation Kit II (MACS, Miltenyi), following manufacturer instructions. CD4+ cells were cultured in RPMI-1640 medium (Gibco) supplemented with 10% Fetal Calf Serum, 2 mM glutamine and antibiotics (100 U/ml penicillin, 100 mg/ml streptomycin). Cells were activated for two days with 100 U/ml IL2 and 5 µg/ml PHA (both from Sigma-Aldrich) and then cultured for 11–14 days in decreasing doses of IL-2 (from 100 U/ml to 10 U/ml).

### Constructs, viral production and infection

HIV-derived vectors were produced by CaPO_4_-mediated transient co-transfection of the lentiviral vector, gag-pol (pCMV. Δ8.74) and VSV-G (pMD2.VSV-G) encoding constructs and titered on HEK 293T cells, as previously described (http://tronolab.epfl.ch/; with minor modifications). For shRNA expression the following puromycin resistance-encoding vectors were used: pLKO.1.shA3G (TRCN0000052188 clone ID:NM_021822.1-398s1c1; Sigma-Aldrich) and pRDI292.shA3Gmismatched [21]. For Vif expression the pRRLsin.cPPT.PGK.GFP-2A-Vif-myc and pRRLsin.cPPT.PGK.GFP-2A-C133SVif-myc vectors were used [21]. Activated CD4+ T cells were exposed to vectors at a multiplicity of infection of 3 for an overnight, washed and reseeded in fresh medium. When using shRNA-expressing vectors, transduced cells were then selected for three days by using 1.5 µg/ml puromycin (Sigma-Aldrich). When using Vif expressing vectors transduced cells were sorted by flow cytometry for GFP expression 3 days after transduction to perform Western blot or gated on the GFP+ population when performing the analysis of the single round infection assay.

As fluorescent protein-expressing HIV reporter virus the pRRLsin.cPPT.PGK.GFP.Wpre [22] or the pRRLsin.cPPT.PGK.CFP.Wpre (kindly provided by Prof. L. Naldini, San Raffaele Telethon Institute for Gene therapy, Milan, Italy) vectors were used. Single round infection assay was performed by exposing the cells to either vector at an MOI of 3 for an overnight, washing and reseeding them in fresh medium. Cells were analyzed 3 days after infection by flow cytometry.

### Flow Cytometry

The following human-specific conjugated antibodies were used: fluorescein isothiocyanate (FITC)-CD4 (Dako), allophycocyanin (APC)-CD25 and phycoerythrin (PE)-CD69 (both from BD Biosciences). To set gates either isotype controls or untransduced cells were used. At least 10000 events per sample were analyzed on Cyan flow cytometer (Dako).

### Western blot

Total proteins were extracted in a radioimmune precipitation assay buffer (phosphate-buffered-saline (PBS) with 1% NP-40, 0.5% sodium deoxycholate and 0.1% SDS) supplemented with protease inhibitor cocktail (Calbiochem). Equal amounts of proteins were resolved on a 10% Bis-Tris NuPage (Invitrogen) gel followed by Western blot. A3G was detected by the following polyclonal antibody, obtained through the NIH AIDS Research and Reference Reagent Program, Division of AIDS, NIAID, NIH: Anti-APOBEC3G from Dr. Warner C. Greene [14], followed by a secondary donkey anti-rabbit antibody conjugated to horseradish peroxidase. Proliferating cell nuclear antigen (PCNA) was used as a protein loading control and was detected using a mouse monoclonal antibody (clone PC10, Calbiochem) followed by a secondary sheep anti-mouse antibody conjugated to horseradish peroxidase. A3G band volumes were quantified by ImageQuant software and normalized to PCNA band volumes.

### High Speed Ultracentrifugation

High speed ultracentrifugation was performed as previously described [16] with minor modifications. Briefly, cells were lysed with ice-cold lysis buffer (125 mM NaCl, 50 mM, Hepes, pH 7.4, 0.2% NP40, 1 mM dithiothreitol, 0.1 mM PMSF, protease inhibitor cocktail; Calbiochem) for 30 min and centrifuged at 35,000 rpm (M150SE; Sorvall) for 1.5 h. SN and P were separated, and P was resuspended in a volume equal to that of SN. Equal volumes of P and SN were loaded on gel and analyzed by Western blot (see above).
